# Effects of Fatigue on Peak and Angle-Specific Hamstring-to-Quadriceps Ratios in Collegiate Women Lacrosse Athletes

**DOI:** 10.3390/jfmk11040424

**Published:** 2026-10-16

**Authors:** Tianyi Lu, Marvin Powell, B. Christine Green, Margaret T. Jones

**Affiliations:** 1 Patriot Performance Laboratory, Frank Pettrone Center for Sports Performance, George Mason University, Fairfax, VA 22030, USA; tlu7@gmu.edu (T.L.); bgreen21@gmu.edu (B.C.G.); 2 Kinesiology, Sport, and Hospitality Management, George Mason University, Fairfax, VA 22030, USA; 3 College of Education and Human Development, George Mason University, Fairfax, VA 22030, USA; mpowel11@gmu.edu

**Keywords:** fatigue, isokinetic testing, quadriceps, hamstring, injury prevention

## Abstract

**Background:** Hamstring strains and anterior cruciate ligament (ACL) tears commonly occur in court and field sport athletes, with prevalence being higher in females. The likelihood of such injuries has been related to the hamstring-to-quadriceps (H:Q) strength ratio. Such a ratio can be affected by fatigue, to which athletes are frequently exposed. The aim of the current study was to investigate effects of fatigue on peak torque and angle-specific functional H:Q ratios at different angular velocities, as well as the association between resting functional H:Q ratios and those obtained following fatigue. **Methods:** A total of 18 collegiate women lacrosse players executed maximal knee concentric and eccentric contractions at angular velocities of 60, 180, and 240°/s following a supervised fatigue protocol. The isokinetic testing was then replicated. **Results:** There was a significant difference in functional peak torque H:Q ratios (*p* = 0.021–0.041) and ratios at 60 (*p* = 0.013–0.044), but not 20 degrees of knee flexion before and after fatigue at all three angular velocities. No main effects of angular velocity or ratio type were observed on H:Q ratio difference. The relationship between resting and fatigued functional H:Q ratios ranged from moderate to strong across all three angular velocities. **Conclusions:** Fatigue was associated with reductions in functional H:Q ratios, indicating altered knee flexor–extensor strength balance under fatigued conditions. In time-constrained settings, baseline functional H:Q ratio may provide practitioners with useful preliminary information regarding an athlete’s potential susceptibility to lower-limb injury after fatigue.

## 1. Introduction

Injuries represent a major concern in sports, as they lead to losses of training and practice time which can compromise performance [1,2]. Hamstring strains and torn anterior cruciate ligaments (ACLs) are two common injuries sustained by athletes from sports which involve running, jumping, and abrupt changes in speed and direction [3,4]. ACL injuries can result in substantial time loss, prolonged rehabilitation, and an incomplete return to preinjury or competitive sport participation [5,6]. Prior research indicates that hamstring strains occur more frequently during the terminal swing phase of sprinting when the hamstrings undergo an intense eccentric action to decelerate knee extension and hip flexion prior to foot contact [7,8,9]. Hamstring muscles are thought to assist the ACL in countering anterior tibial drawer forces during leg extension by enhancing the posterior pull, solidifying joint stiffness, and alleviating anterior laxity ligament force under knee stress [10,11]. Further, co-activation of the hamstrings and quadriceps during extension reduces the tensile stress on the ACL compared to activating quadriceps alone, highlighting the importance of hamstring strength in ACL injury prevention [12,13,14].

The hamstring-to-quadriceps (H:Q) strength ratio has become a pivotal focus in sports medicine and biomechanics research due to its implications for muscle imbalance identification, knee joint stability assessment, characterization of tissue property and functionality, and injury reduction and rehabilitation [15,16]. The H:Q ratio is typically derived from isokinetic torque measurements, where torque represents the rotational force generated by the knee flexors and extensors around the joint axis [17]. The conventional H:Q ratio represents the ratio of hamstring concentric peak torque to quadriceps concentric peak torque measured at the same angular velocity during isokinetic testing [18]; however, its clinical utility may be limited because it does not accurately represent the opposing muscle actions that occur during dynamic sport movements [19,20]. Divr and colleagues [21] introduced the concept of the functional H:Q ratio, or the “dynamic control” ratio, which is defined as the ratio of hamstring eccentric peak torque to quadriceps concentric peak torque at the same angular velocity. A functional H:Q ratio of 1 between the hamstring and quadricep muscles is considered desirable across a variety of isokinetic angular velocities and knee angles, when the actual ratio exhibits a positive correlation with the angular velocity [22,23].

Fatigue is an important consideration when interpreting H:Q ratios because sport-related lower-limb injuries often occur under conditions of repeated high-intensity activity [24,25]. Women athletes experience ACL tears at a higher rate than male athletes, especially in court and field sports [26]. In part, this may be attributed to the lower functional H:Q ratio observed in females [23,27,28,29,30]. Also, short bursts of intense activity, which cause transient fatigue, in conjunction with prolonged, cumulative fatigue throughout the duration of a game increase the likelihood of hamstring and ACL injuries [31,32]. When a fatigue protocol of high-repetition isokinetic contractions was implemented in age- and health-status-matched males and females, both groups exhibited a lower functional H:Q ratio post-fatigue, with a greater magnitude of decrement observed in females [30]. Therefore, examining functional H:Q ratios under fatigued conditions may provide a more sport-relevant understanding of knee joint strength balance than assessments performed in a rested state.

The joint-angle-specific H:Q ratio, defined as the H:Q torque ratio at a particular knee angle throughout the entire range of motion, has served as an alternative method for assessing knee joint strength balances, as the peak torque values typically occur at non-matching antagonist–agonist angles [15,33,34,35]. This ratio is used as an indicator of knee joint stability, particularly under high-velocity conditions where the antagonist must eccentrically counteract the momentum of agonist-driven movements at the end of the joint range [15,33,34,35]. However, data on the joint-angle-specific H:Q ratio is limited.

Although H:Q ratios are frequently discussed in relation to injury screening, their ability to independently predict ACL or hamstring injuries remains uncertain. Kellis et al. systematically reviewed prospective evidence and concluded that H:Q ratios, including conventional and functional ratios, have limited value as stand-alone predictors of ACL or hamstring injury [36], whereas Dauty et al. suggested that the predictive value of isokinetic ratios may depend upon the calculation method used [37]. Therefore, in the present study, functional H:Q ratios are interpreted as indicators of knee flexor–extensor strength balance, rather than as direct measures of injury susceptibility.

Despite previous work examining fatigue-related changes in functional H:Q ratios, limited evidence is available regarding how a field-based fatiguing protocol affects both peak torque and angle-specific functional H:Q ratios in women athletes. Furthermore, it remains unclear whether resting functional H:Q ratios are associated with post-fatigue functional H:Q ratios in this population. Therefore, the purpose of the current study was to examine the impact of fatigue-eliciting exercises on peak torque and angle-specific H:Q ratios across various angular velocities. A second purpose was to assess the relationship between the resting functional H:Q ratio and the functional H:Q ratio following fatigue in collegiate women athletes. It was expected that the fatigue protocol would be associated with reductions in functional H:Q ratios. Because previous work suggests that fatigue-related changes in functional H:Q ratios may vary across angular velocities, we also explored whether the magnitude of change differed by angular velocity. However, given the limited evidence comparing peak and angle-specific functional H:Q ratios following field-based fatigue in collegiate women athletes, the comparison across ratio types was considered exploratory. Additionally, a positive relationship would be expected between the resting H:Q ratio and the ratio following fatigue.

## 2. Materials and Methods

### 2.1. Participants

A convenience sample of 18 National Collegiate Athletic Association Division I (NCAA DI) women lacrosse athletes participated. The sample size was determined by the availability of eligible NCAA Division I women lacrosse athletes during the data collection period. Therefore, an a priori calculation of sample size was not performed. All athletes maintained regular training routines aligned with the physical and neuromuscular requirements of their competitive sport season. Inclusion and exclusion criteria were followed to maintain data integrity and the applicability of the outcomes. Athletes were 18–28 years of age and free of chronic illness and lower-limb injuries within the previous 6 months. Pregnancy, as self-identified, was an exclusion criterion. Procedures were performed in compliance with the ethical standards established in the Declaration of Helsinki and approved by the University Institutional Review Board (project code: 1668426-3). Written informed consent was obtained from all athletes for use of their testing results in research. All data were de-identified to preserve privacy and confidentiality.

### 2.2. Procedures

Athletes were instructed to abstain from engaging in physically demanding activities for 48 h before reporting to the laboratory. Such activities included resistance training, endurance running, strenuous hiking, competitive sports, and occupations involving repetitive heavy lifting. A supervised, standardized 10-min warm-up consisting of countermovement jumps, squats, lunges, and dynamic stretching was performed prior to testing. Countermovement jumps (CMJ) were conducted via portable bilateral force plates (VALD, Brisbane, Queensland, Australia). Knee joint isokinetic assessments were performed using calibrated isokinetic dynamometry (HUMAC NORM, CSMI, Stoughton, MA, USA).

**Baseline Testing:** Following the warm-up, athletes performed three maximal-effort CMJs with hands on hips across all trials. Athletes were instructed to jump as fast and as high as possible following a countermovement to a self-selected depth on the count of “3, 2, 1, JUMP!” while securing and holding their landing position. After the jumps, athletes were positioned comfortably in the isokinetic dynamometer chair by the same trained researcher. The hip and knee joints were flexed to approximately 85°, with 0° representing full knee extension—a positioning conventionally employed in isokinetic testing to optimize mechanical advantage and joint stabilization [38]. Full knee extension was visually confirmed and set as 0°, after which the dynamometer was used to determine the 85° knee flexion position. The dominant leg, defined operationally as the limb preferentially used for executing dynamic tasks such as kicking a ball, was carefully affixed to the lever arm of the dynamometer. The distal shin pad was positioned 2–3 cm proximal to the lateral malleolus, with adjustment performed using a non-elastic strap. To mitigate extraneous movements and ensure that the force output originated exclusively from the musculature under investigation (i.e., quadriceps and hamstrings), stabilization straps were applied to the chest, pelvis, and mid-thigh. Alignment between the dynamometer’s mechanical axis of rotation and the anatomical axis of knee joint was noted for each athlete and rechecked at the onset of each testing condition to ensure accuracy and reliability of torque data. Testing was conducted through a 90° to 5° range of motion, with gravity correction performed using the system’s proprietary software [39]. Next, athletes underwent a familiarization protocol consisting of three submaximal concentric extensions at increasing intensities (50%, 75%, 90%). Subsequently, they executed three continuous maximal concentric and eccentric contractions at angular velocities of 60, 180, and 240°/s, with consistent verbal encouragement provided throughout by the same trained researcher.

**Fatigability Protocol:** Following the baseline testing, athletes performed a fatigability protocol as follows: Researchers calculated and marked 80% of each athlete’s maximal CMJ on the force plates. Athletes then performed the activities within a fatigue protocol of four exercises performed consecutively upon completion, similar to that illustrated in previous research [40]. Exercises were performed in the following order: 10 bodyweight squats to at least 90° of knee flexion, five single leg non-countermovement jumps (SLNCMJ) with dominant leg onto a standard 6-inch box, a 20-yard shuttle run, and 3 CMJs. Close observation was provided throughout the fatigue protocol to ensure quality movement and appropriate effort. If a new CMJ height was greater than the 80% fatigue threshold, the fatigue protocol would be performed again. Once CMJ fell below 80% on 3 consecutive attempts, the fatigue protocol was terminated. This criterion-based termination approach was used to individualize the fatigue stimulus relative to each athlete’s own CMJ performance rather than requiring all athletes to complete a fixed number of cycles. As athletes differ in fitness, neuromuscular capacity, and fatigue resistance, the number of cycles required to reach the 80% CMJ threshold was expected to vary across participants. After the fatigability protocol, athletes replicated the baseline isokinetic testing without additional warm-up trials. Post-fatigue isokinetic testing was initiated as soon as possible after termination of the fatigue protocol. The fatigue protocol was performed less than 10 feet from the isokinetic dynamometer, allowing athletes to transition immediately back to the dynamometer chair for repositioning and stabilization. Baseline and post-fatigue isokinetic testing were performed in the same fixed ascending order of angular velocity: 60°/s, 180°/s, and 240°/s. This fixed ascending order was used to maintain procedural consistency across athletes and between baseline and post-fatigue assessments. Previous fatigue studies have similarly examined pre- and post-fatigue H:Q ratios across multiple angular velocities [30,41] In addition, ascending velocity orders and standardized between-velocity rest intervals have been used in common isokinetic strength-testing protocols, with prior work suggesting that at least 60 s between velocity sets is sufficient for recovery during multi-velocity isokinetic testing [42].

The joint angles of 20°, 40°, and 60° of knee flexion were selected to examine angle-specific functional H:Q ratios across the tested range of motion. The 20° position was selected as the closest angle to terminal knee extension that could be consistently evaluated across participants and angular velocities, whereas 40° and 60° represented progressively more flexed knee positions. The functional H:Q ratio obtained for the maximum contraction across three distinct velocities (60°/s, 180°/s, and 240°/s) and at each specified joint angle, along with the peak functional H:Q ratio, were calculated for analysis purposes using the following formulas:

$$\mathrm{Peak}\;\mathrm{functional}\;H:Q\;\mathrm{ratio}\;=\;\frac{\mathrm{Hamstring}\;\mathrm{eccentric}\;\mathrm{peak}\;\mathrm{torque}\;}{\mathrm{Quadriceps}\;\mathrm{concentric}\;\mathrm{peak}\;\mathrm{torque}\;}$$

$$\mathrm{Functional}\;H:Q\;\mathrm{ratio}\;\mathrm{at}\;20{}^{\circ}\;\mathrm{knee}\;\mathrm{flexion}\;=\;\frac{\mathrm{Hamstring}\;\mathrm{eccentric}\;\mathrm{torque}\;\mathrm{at}\;20{}^{\circ}}{\mathrm{Quadriceps}\;\mathrm{concentric}\;\mathrm{torque}\;\mathrm{at}\;20{}^{\circ}\;}$$

$$\mathrm{Functional}\;H:Q\;\mathrm{ratio}\;\mathrm{at}\;40{}^{\circ}\;\mathrm{knee}\;\mathrm{flexion}\;=\frac{\mathrm{Hamstring}\;\mathrm{eccentric}\;\mathrm{torque}\;\mathrm{at}\;40{}^{\circ}}{\mathrm{Quadriceps}\;\mathrm{concentric}\;\mathrm{torque}\;\mathrm{at}\;40{}^{\circ}\;}$$

$$\mathrm{Functional}\;H:Q\;\mathrm{ratio}\;\mathrm{at}\;60{}^{\circ}\;\mathrm{knee}\;\mathrm{flexion}\;=\frac{\mathrm{Hamstring}\;\mathrm{eccentric}\;\mathrm{torque}\;\mathrm{at}\;60{}^{\circ}}{\mathrm{Quadriceps}\;\mathrm{concentric}\;\mathrm{torque}\;\mathrm{at}\;60{}^{\circ}\;}$$

### 2.3. Statistical Analysis

Statistical analyses were conducted using the Statistical Package for the Social Sciences (SPSS; Version 24.0; IBM Corp., Armonk, NY, USA). Descriptive statistics (mean ± SD) including age, height, and body mass were calculated to characterize the sample population. Paired-sample *t*-tests were employed to compare pre- and post-fatigue values for each ratio type. Subsequently, a 3 (angular velocity) × 4 (H:Q ratio type) repeated-measures analysis of variance (ANOVA) was performed. An alpha level of 0.05 was set a priori for significance, and a post hoc Bonferroni adjustment was employed when a main effect was present. Effect sizes for paired-sample *t* tests were calculated using Cohen’s d, with thresholds set at 0.2 for small effects, 0.5 for medium effects, and 0.8 for large effects [43]. A convenience sample was used in the current study; therefore, descriptive statistics and effect sizes were emphasized to characterize the magnitude and direction of fatigue-related changes, while *p* values indicated relationships within the sample rather than making inferences about the larger population of athletes. Partial eta-squared (η^2^_p_) was calculated to quantify effect sizes for the repeated-measures ANOVA main effects and interaction effects [44]. Pearson’s r was utilized to examine the linear correlation between pre- and post-fatigued functional H:Q ratios, with thresholds set at 0–0.3 for small, 0.3–0.7 for moderate, and 0.7–1.0 for strong correlations [45].

## 3. Results

Descriptive statistics of demographic variables are presented in Table 1.

Differences were found in functional peak torque H:Q ratios at all angular velocities (60°/s: *p* = 0.041, d = 0.36; 180°/s: *p* = 0.021, d = 0.40; 240°/s: *p* = 0.025, d = 0.41) before and after fatigue. Further, differences in functional H:Q ratios before and after fatigue occurred at 60° of knee flexion for angular velocities of 60°/s (*p* = 0.044, d = 0.29), 180°/s (*p* = 0.013, d = 0.49), and 240°/s (*p* = 0.022, d = 0.50). The difference in functional H:Q ratios at 40° of knee flexion before and after fatigue was significant at an angular velocity of 240°/s (*p* = 0.038, d = 0.37). Functional H:Q ratios at 20° of knee flexion before and after fatigue did not differ an any angular velocity (Table 2).

There were no main effects of angular velocity (*p* = 0.116, $\eta_{p}^{2}$ = 0.021) or H:Q ratio type (*p* = 0.709, $\eta_{p}^{2}$ = 0.007) on the H:Q ratio differences (Table 3).

**Figure 1:**
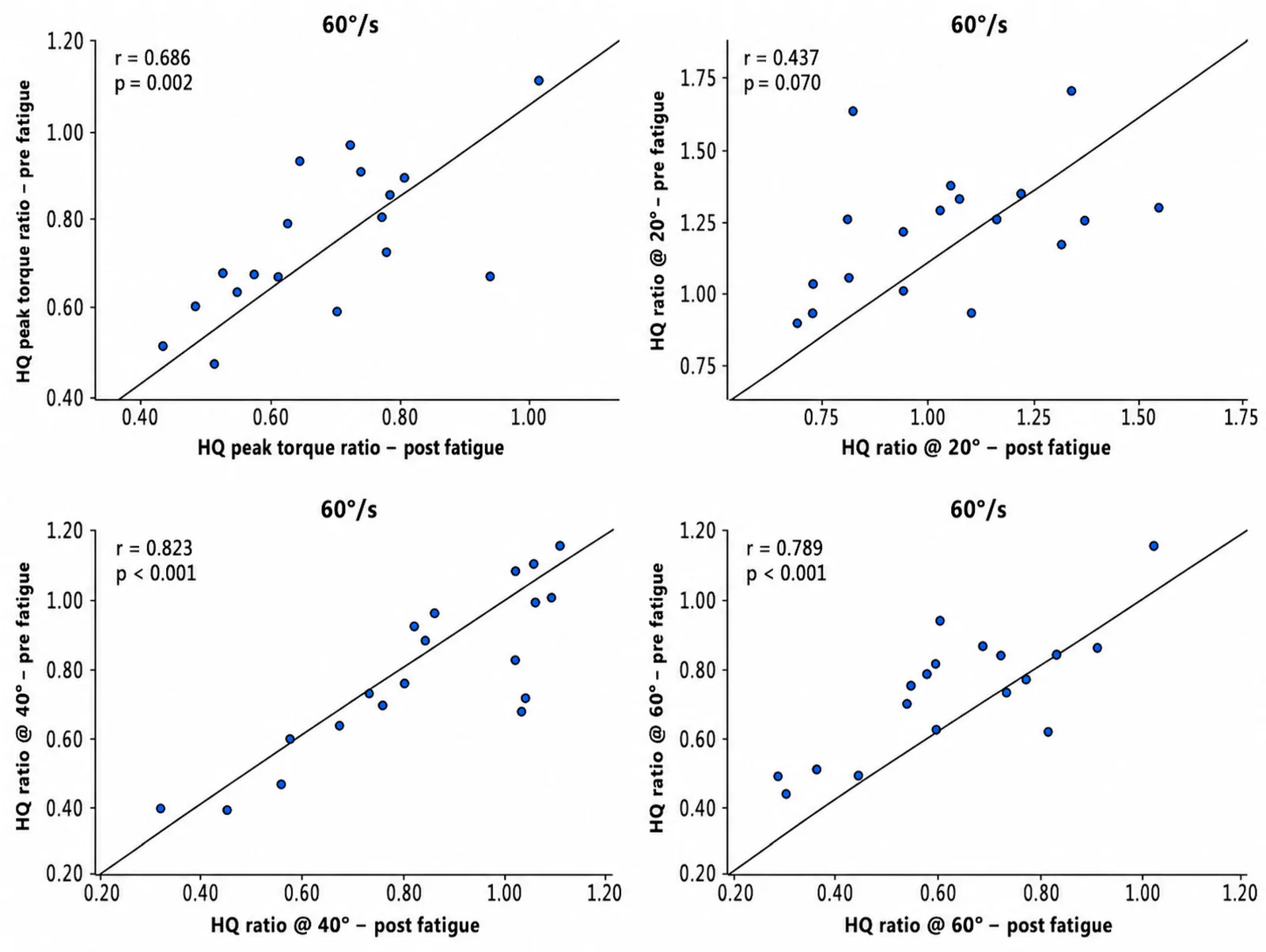
Pearson’s r correlations between different types of functional H:Q ratios at baseline (before fatigue) and after fatigue at 60°/s angular velocity.

**Figure 2:**
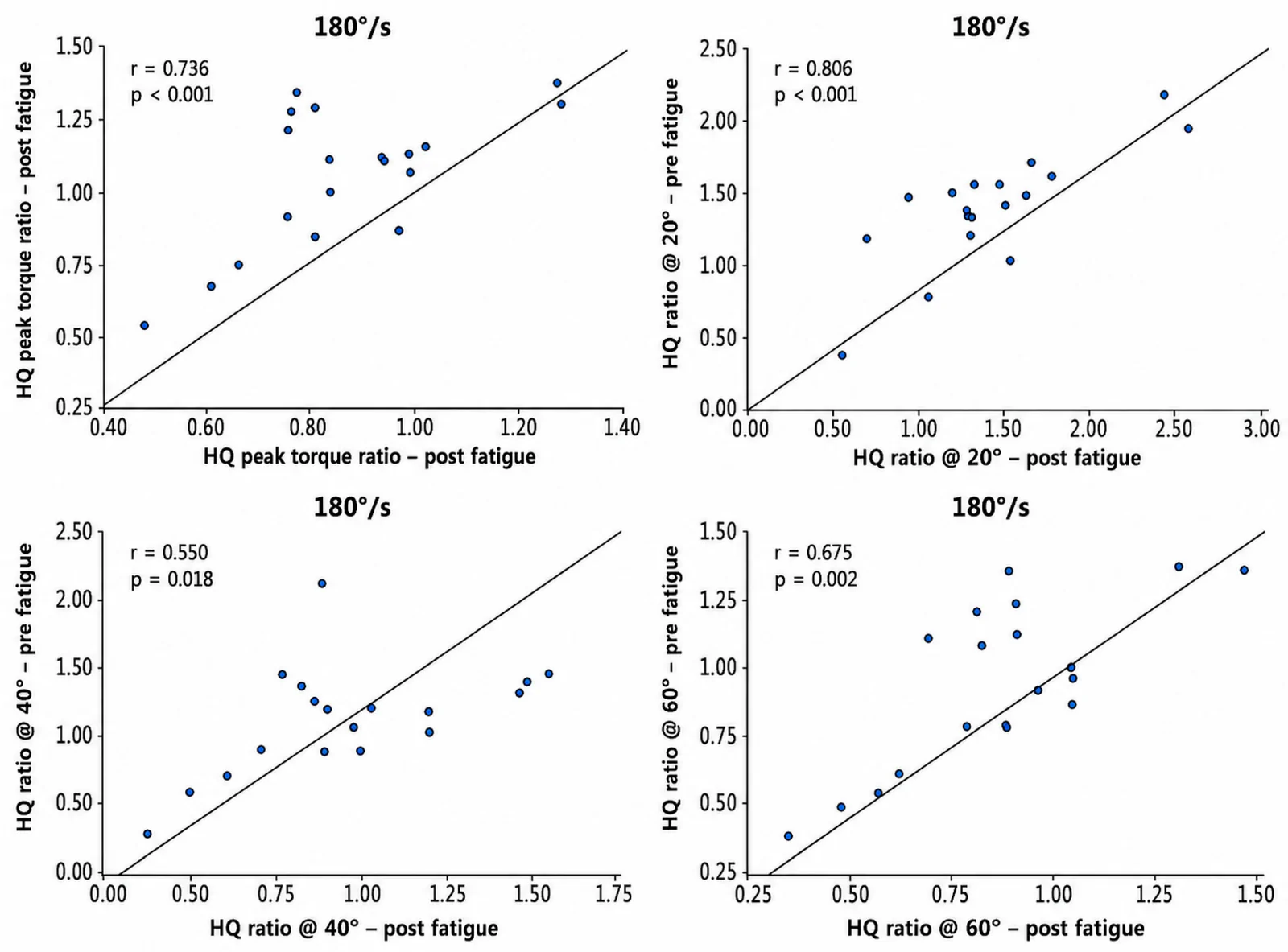
Pearson’s r correlations between different types of functional H:Q ratios at baseline (before fatigue) and after fatigue at 180°/s angular velocity.

**Figure 3:**
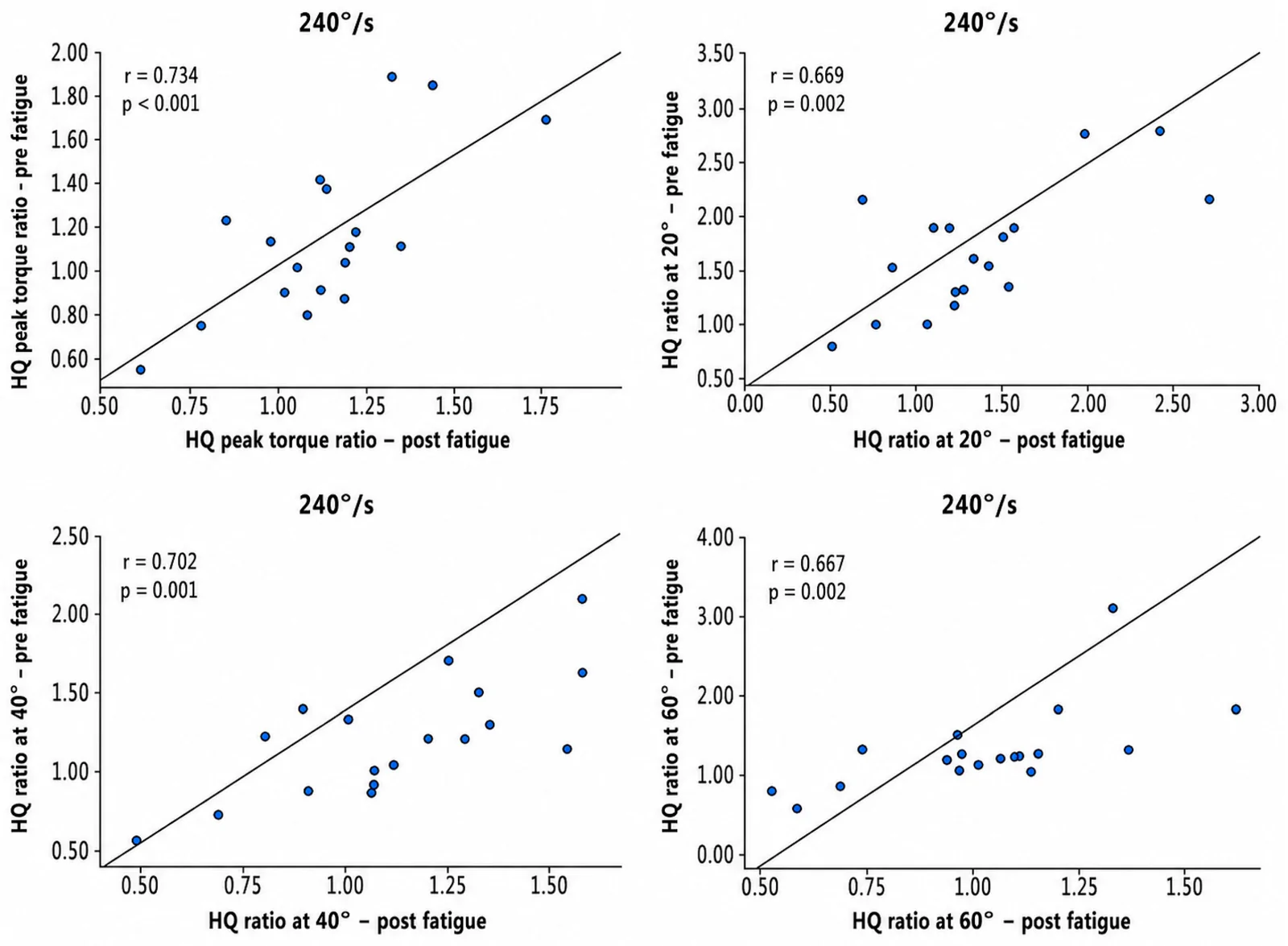
Pearson’s r correlations between different types of functional H:Q ratios at baseline (before fatigue) and after fatigue at 240°/s angular velocity.

Regression lines are included in Figure 1, Figure 2 and Figure 3 to visually illustrate the direction and magnitude of the linear associations between baseline and post-fatigue functional H:Q ratios.

Figure 1 demonstrates the relationship between baseline and post-fatigue functional H:Q ratio at 60°/s. Figure 2 demonstrates the relationship between baseline and post-fatigue functional H:Q ratio at 180°/s. Figure 3 demonstrates the relationship between baseline and post-fatigue functional H:Q ratio at 240°/s. Pearson’s r values showed that the relationship between resting and fatigued functional H:Q ratios remained moderate to strong across all three angular velocities.

## 4. Discussion

To our knowledge, this is the first investigation to systematically assess the impact of fatigue on peak torque and angle-specific functional H:Q ratios at slow and fast angular velocities in women collegiate lacrosse players. In addition, we assessed whether resting functional H:Q ratios were associated with post-fatigue ratios following a field-based fatiguing protocol. Overall, the findings of the present investigation reaffirmed the decline in functional H:Q ratio after fatigue previously reported for other populations (e.g., active adults, male soccer athletes) [30,31,32,34]. In recent years, the functional H:Q ratio has been used to characterize the eccentric capacity of the hamstrings as the antagonist muscle group during knee extension. Because this ratio reflects opposing muscle actions that occur during dynamic movement, it may provide useful information regarding knee flexor-–extensor strength balance [46].

Injury incidence tends to escalate during the later stages of athletic performance, particularly in sports with prolonged duration, and this is frequently attributed to fatigue-impaired neuromuscular function [47,48]. Fatigue-related reductions in hamstring torque may be particularly important because the hamstrings contribute to dynamic knee stabilization by counteracting quadriceps-driven anterior tibial translation. However, recent prospective evidence has questioned the ability of H:Q ratios to independently predict ACL or hamstring injuries, stating that H:Q ratios have limited value as stand-alone injury-prediction measures and should be interpreted alongside other biomechanical, neuromuscular, and sport-specific risk factors [36]. Therefore, the current findings are best interpreted as fatigue-related alterations in knee flexor–extensor strength balance in collegiate women lacrosse athletes.

A primary finding of the current study was that significant differences in peak torque functional H:Q ratios and functional ratio at 60° of knee flexion were identified before and after fatigue, independent of angular velocity, whereas the ratios at 20° remained statistically unchanged. A significant difference in the functional H:Q ratio difference at 40° of flexion occurred solely under the 240° condition. The magnitude of the observed changes was generally small-to-moderate. Peak torque functional H:Q ratios decreased by 6.1%, 10.2%, and 12.6% at 60°/s, 180°/s, and 240°/s, respectively, with Cohen’s *d* values ranging from 0.36 to 0.41. Similarly, fatigue-related changes at 60° of knee flexion ranged from 5.6% to 22.3%, with effect sizes ranging from 0.29 to 0.50, indicating that fatigue-related changes were generally small-to-moderate but directionally consistent across velocities. These patterns in peak torque functional H:Q ratio changes supported previous findings [41,49,50]. When male amateur and professional soccer players were tested at a variety of angular velocities, their functional peak torque H:Q ratio decrement ranged from 14.3–29.8% at speeds from 120 to 300°/s, a more substantial value than that of the conventional ratio [49]. While the fatigue protocol employed was treadmill running, absent the high-speed sprinting and directional shifts and running patterns existing in an actual game, the declines were larger than those of the current study (6.0–12.6) [49]. Additional research in which male semi-professional soccer players had their extension and flexion torque measured before and after multidirectional soccer-specific field tests with frequent acceleration and deceleration reported that the functional H:Q ratio exhibited a 16.8% decrease at 120°/s [50]. A comparably constructed study with male soccer athletes conducted to examine the change in strength relationship under two speeds showed that the functional H:Q ratio decreased by 17.7% (79% vs. 65%) at 60 deg/sec and 14.4% (90% vs. 70%) at 180°/s post-fatigue [41]. In another study in which the same fatigue protocol was implemented, involving age- and health-status-matched male and female physically active participants, both groups exhibited a significantly lower functional H:Q ratio post-fatigue, but the magnitude of decrement was more profound in females [30]. Specifically, the ratio reductions recorded for the females seem to be more pronounced than those determined for female athletes in the current investigation. The smaller magnitude of reductions in the collegiate women lacrosse population in the current work may be partly explained by the nature and duration of the fatigue protocol used. Participants stopped the protocol once CMJ height fell below 80% for three consecutive attempts, a criterion implemented partly as a precautionary measure to protect athletes from excessive fatigue or injury. Therefore, the protocol may not have produced the same level of neuromuscular exhaustion as longer match-play or soccer-specific protocols involving repeated sprinting and multidirectional running. In addition, differences in sport population, training background, and movement demands between collegiate women lacrosse players and male soccer players may have contributed to the smaller observed decrements. Future studies with larger, prospectively powered samples should determine whether these small-to-moderate effects are reproducible and whether the magnitude of change differs meaningfully across angular velocities or joint-angle-specific ratio measures.

The incidence of ACL and hamstring injuries is disproportionately higher among female athletes compared to their male counterparts [26]. It is believed that this may be due to multiple interacting factors, including quadriceps-dominant movement patterns. In fact, an array of studies have shown that females have higher quadriceps muscle activation than males during functional movements [51,52,53,54,55,56,57], as well as differences in hormonal response [58,59], femoral notch width and shape [60,61], and core stability [26,62]. Therefore, the more subtle ratio differences observed in the current study may be partly attributed to differences in fatigue-protocol duration. The aforementioned study employed a longer protocol designed to replicate the demands of actual match play. This likely produced a greater level of fatigue before post-fatigue isokinetic testing than what was observed in the women lacrosse players in the current study.

A cross-sectional study with female soccer athletes examined the H:Q ratio in response to fatigue and reported an unchanged conventional and increased functional H:Q ratio (+11.3%) [63]. It was proposed this might be due to the fatigue protocol being a bout of high-repetition isokinetic burnout concentric-only contractions, as opposed to a balanced dynamic routine [63,64]. Evidence from prior research indicates that eccentric strength deteriorates to a larger extent when preceded by eccentric, rather than concentric, exercise [65]. Overall, fatiguing exercises seemingly contribute to force-generating capacity change in both quadriceps and hamstrings, but particularly in hamstrings. To explain, previous studies have illustrated several potential theories, including possession by the hamstring of a higher percentage of fast-twitch muscle fibers that are more dependent on the anaerobic metabolism and prone to glycogen depletion [33,66,67,68], a larger optimal eccentric peak torque production shift toward shorter muscle lengths during exercise [50], and less engagement in daily activities, compared to quadriceps [64], although alternative mechanisms may remain unknown. It is suggested that future investigations are designed to analyze sex differences in the effect of dynamic fatigue on the H:Q ratio in a controlled environment, perhaps among a variety of field and court sport athletes.

Secondary findings from the current study indicate that neither functional H:Q ratio type nor angular velocities affected the magnitude of fatigue-related ratio decrement. This finding differs partially from previous work with physically active adults by El-Ashker and colleagues, who reported that fatigue may have a greater effect on torque production at lower degrees of knee flexion; however, significant differences in the H:Q ratio change were observed only at 15° compared to 30° and 45° of knee flexion at 300°/s, whereas ratio changes at 60°/s and 180°/s did not differ [30]. Similarly, another study reported a decline in the functional H:Q ratio at 10° of knee flexion, while ratios between 90° and 20° remained unchanged [41]. Therefore, it is possible that significant angle-specific differences might have emerged in the current study if ratios had been examined at 15° of knee flexion or closer to terminal knee extension. However, testing at these end-range positions may be challenging, particularly at higher angular velocities, because athletes may have difficulty maintaining the preselected speed near the end range of knee motion. It was previously reported that greater angular velocities elicited larger reductions in functional H:Q ratios [30]. The final finding demonstrates that the resting functional H:Q ratio was moderately to strongly associated with post-fatigue ratios. This relationship indicates that athletes with relatively higher functional H:Q ratios at baseline tended to maintain relatively higher values following fatigue. However, this association should not be interpreted as evidence of predictive validity, because the analysis did not determine whether baseline H:Q ratios identified which athletes experienced greater fatigue-related deterioration. From an applied perspective, this finding suggests that resting functional H:Q ratios may provide practitioners with useful preliminary information regarding post-fatigue knee strength balance when time constraints limit more extensive fatigue-based testing, and thus guide decisions regarding further assessment, injury-prevention programming, or individualized strength interventions.

The current study is not without limitations. Firstly, the fatigue protocol had athletes cease participation when CMJ fell below only 80% on three consecutive attempts. Although the protocol included field-sport-relevant movements such as squatting, unilateral jumping, shuttle running, and repeated CMJs, it might not have fully reproduced neuromuscular exhaustion in game-specific conditions. The decision was made as part of precautionary measures to protect athletes from potential injuries and limit excessive fatigue during the competitive season. Secondly, the study used a convenience sample of 18 collegiate women lacrosse athletes, and no a priori calculation of sample size was performed. Therefore, the sample may not be representative of all collegiate women field-sport athletes, and the study may have limited precision for detecting effects related to angular velocity or ratio type. An additional limitation is that the current dataset only comprises collegiate women lacrosse athletes. This homogeneity in athletic background may limit the generalizability of the findings to specific sporting populations, as sport-specific neuromuscular and biomechanical demands tends to differ across sport and events.

## 5. Conclusions

The present investigation aimed to systematically examine the influence of fatigue induced via standardized field-based protocol on peak torque and angle-specific functional H:Q ratios across slow and fast angular velocities. Additionally, we sought to evaluate the relationship between resting functional H:Q ratios and post-fatigue values in collegiate women lacrosse athletes. Statistical analysis revealed a significant decline in the functional H:Q peak torque ratios and those measured at 60° of knee flexion across all three angular velocities following fatigue. Further, the association between resting and fatigued functional H:Q ratios appeared to be moderate to strong. These findings suggest that fatigue alters knee flexor–extensor strength balance in collegiate women lacrosse athletes. Practitioners working in time-constrained settings may use baseline functional H:Q ratios to obtain preliminary insight into post-fatigue strength balance; however, these ratios should not be used as stand-alone indicators of injury risk or as indicators of which athletes will experience greater fatigue-related deterioration.

## Figures and Tables

**Table 1:** Descriptive Statistics (*n* = 18).

Variable	Mean	±SD
Age (yrs.)	19.46	1.22
Body Height (cm)	165.59	5.12
Body Mass (kg)	65.16	5.75

Note: SD = standard deviation; yrs = years; cm = centimeters; kg = kilograms.

**Table 2:** Comparison of functional H:Q ratio (%) before and after fatigue at three angular velocities.

Angular Velocity	Functional H:Q Torque Ratio	Before Fatigue		After Fatigue		*p* Value	Cohen’s d
		Mean	SD	Mean	SD		
60°/s	peak	72.6	18.0	66.5	15.7	0.041	0.36
	20	112.4	25.5	105.2	26.6	0.149	0.28
	40	84.0	19.3	84.1	22.6	0.484	0.00
	60	67.2	19.3	61.6	19.9	0.044	0.29
180°/s	peak	98.3	27.9	88.1	22.4	0.021	0.40
	20	134.1	43.3	130.6	52.3	0.321	0.07
	40	109.0	41.5	95.1	27.2	0.060	0.40
	60	93.7	30.1	80.6	22.5	0.013	0.49
240°/s	peak	116.1	36.3	103.4	24.8	0.025	0.41
	20	144.8	49.2	133.3	60.0	0.157	0.21
	40	120.4	38.5	107.7	29.7	0.038	0.37
	60	118.9	56.1	96.6	29.7	0.022	0.50

Note: SD = standard deviation; peak = peak functional H:Q ratio; 20 = functional H:Q ratio at 20° of knee flexion; 40 = functional H:Q ratio at 40° of knee flexion; 60 = functional H:Q ratio at 60° of knee flexion. Statistical significance was set at *p* = 0.05. The paired-comparison *p* values were unadjusted and are presented descriptively.

**Table 3:** Comparison of functional H:Q ratio difference (%) before and after fatigue across three angular velocities.

Ratio Type	Variable	60 deg/s	180 deg/s	240 deg/s	Int. (η^2^_p_)	Type. (η^2^_p_)	Velocity. (η^2^_p_)
Peak	Mean ± SD	6.1 ± 13.9	10.2 ± 19.5	12.6 ± 25.4	0.885 (0.011)	0.709 (0.007)	0.116 (0.021)
	95% CI	−7.4–19.5	−3.3–23.7	−0.9–26.1			
20°	Mean ± SD	7.2 ± 28.4	3.6 ± 31.9	11.5 ± 46.8			
	95% CI	−6.3–20.7	−9.9–17.1	−2.0–25.0			
40°	Mean ± SD	−0.1 ± 13.2	13.8 ± 35.9	12.7 ± 28.4			
	95% CI	−13.6–13.4	0.4–27.4	−0.8–26.2			
60°	Mean ± SD	5.6 ± 13.1	13.1 ± 22.9	22.3 ± 43.6			
	95% CI	−7.9–19.1	−0.4–26.6	8.8–35.8			

Note: SD = standard deviation; CI = confidence interval; peak = H:Q peak torque ratio difference; 20° = H:Q torque ratio difference at 20° of knee flexion; 40° = H:Q torque ratio difference at 40° of knee flexion; 60° = H:Q torque ratio difference at 60° of knee flexion.

## Data Availability

The data presented in this study are available upon reasonable request from the corresponding author.
